# Insight Into the Superlubricity and Self-Assembly of Liquid Crystals

**DOI:** 10.3389/fchem.2021.668794

**Published:** 2021-06-11

**Authors:** Shanchao Tan, Jiayu Tao, Wendi Luo, Hongyu Shi, Bin Tu, Hao Jiang, Yuhong Liu, Haijun Xu, Qingdao Zeng

**Affiliations:** ^1^State Key Laboratory of Tribology, Tsinghua University, Beijing, China; ^2^Chinese Academy of Sciences (CAS) Key Laboratory of Standardization and Measurement for Nanotechnology, CAS Center for Excellence in Nanoscience, National Center for Nanoscience and Technology, Beijing, China; ^3^Jiangsu Co-Innovation Center of Efficient Processing and Utilization of Forest Resources, College of Chemical Engineering, Nanjing Forestry University, Nanjing, China; ^4^Laboratory of Theoretical and Computational Nanoscience, CAS Key Laboratory of Nanophotonic Materials and Devices, CAS Center for Excellence in Nanoscience, Beijing Key Laboratory of Ambient Particles Health Effects and Prevention Techniques, National Center for Nanoscience and Technology, Chinese Academy of Sciences, Beijing, China; ^5^School of Chemistry and Chemical Engineering, Henan Normal University, Xinxiang, China; ^6^Center of Materials Science and Optoelectonics Engineering, University of Chinese Academy of Sciences, Beijing, China

**Keywords:** nanotribology, superlubricity, self-assembly, liquid crystal, interaction strength

## Abstract

Liquid crystals are promising molecular materials in the application of lubrication. Herein, the microscale solid superlubricity is accomplished by the construction of uniform and ordered self-assembly of several liquid crystals. The self-assembly structures on a highly oriented pyrolytic graphite (HOPG) surface are explicitly revealed by using scanning tunneling microscopy (STM). Meanwhile, the nanotribological performance of the self-assemblies are measured by using atomic force microscopy (AFM), revealing ultralow friction coefficients lower than 0.01. The interaction energies are calculated by density functional theory (DFT) method, indicating the positive correlation between friction coefficients and interaction strength. The effort on the self-assembly and superlubricity of liquid crystals could enhance the understanding of the nanotribological mechanism and benefit the further application of liquid crystals as lubricants.

## Introduction

In the current industrialized world, research on tribology has been quite important to increase the components service life and save energy (Perry and Tysoe, 2005). Hereinto, the new concept superlubricity, which refers to the ultralow friction with negligible energy dissipation, has attracted more and more attention (Hirano and Shinjo, 1993; Hod et al., 2018). The current researches of solid superlubricity mainly focus on the inorganic materials such as molybdenum disulfide (MoS_2_) (Martin et al., 1993), graphite (Mate et al., 1987), and graphene (Ge et al., 2018). Compared to these inorganic solid superlubricity materials, liquid crystals have modifiable functional groups, which makes it possible to tailor unique functionalities and combine them with superlubricity to explore further application. Therefore, the effort on the superlubricity of liquid crystals could extend the research field of solid superlubricity from inorganic to organic and benefit the further application of solid superlubricity.

In essence, the friction process is the macro manifestation of molecular behaviors occurring at the rubbing interface (Filippov et al., 2004; Socoliuc et al., 2004; Schwarz and Holscher, 2016). Therefore, it is of great significance to investigate the tribological behaviors at molecular level. In our previous research on self-assembly of template networks (Shi et al., 2018), we revealed the regular nanostructures induced by hydrogen bond and van der Waals force. Based on the theoretical calculation, it was concluded that the friction coefficients at nanoscale were positively correlated with the interaction strength in supramolecular assembly. And in our research of fullerene derivatives (Tan S. C. et al., 2020), we achieved the microscale superlubricity by the construction of ordered host-guest assembly structures. However, further investigation remains necessary to explore the mechanism of tribology and promote the achievement of superlubricity.

As molecular materials that possess both order and mobility (Bisoyi and Kumar, 2011; Kato et al., 2018), liquid crystals are finding rising applications in a wide variety of fields including liquid-crystal display (LCD) device (Drzaic, 1986; Schadt et al., 1996; Sheraw et al., 2002), catalysis (Xu et al., 2004; Gin et al., 2006), biomedicine (Woltman et al., 2007; Lee et al., 2012), electron and ion transport (Kato et al., 2017; Dong et al., 2019), sensor (Bi et al., 2009; Hussain et al., 2016; Popov et al., 2018), etc. Besides, liquid crystals are also good candidates for lubricant, which have shown their ability to lower the friction coefficients and wear rates of sliding surfaces (Carrion et al., 2009; Usol'tseva and Smirnova, 2019). Gao et al. (2017) investigated the regulation of electrical field on a kind of nematic liquid crystal. In the low-speed range, the liquid crystal molecules could arrange in the direction of the electrical field when the voltage increased and perform a lower friction coefficient, which could be applied in smart bearings. Mokshin (2020) reduced the friction coefficient and near-contact temperature of steel-steel friction pair to about a half by using cholesterol liquid crystal as additives of mineral oil. Chen et al. (2019) synthesized a kind of lamellar liquid crystals by using ionic liquids as cosurfactants. The increase of the concentration of ionic liquids and the length of alkyl chain in the lamellar liquid crystals can lead to better performances in friction-reducing and antiwear, which are promising in water lubrication. However, the current tribological research on liquid crystals mainly focus on the performance at macroscale. The tribological property and mechanism at molecular level are rarely investigated.

**Scheme 1:**
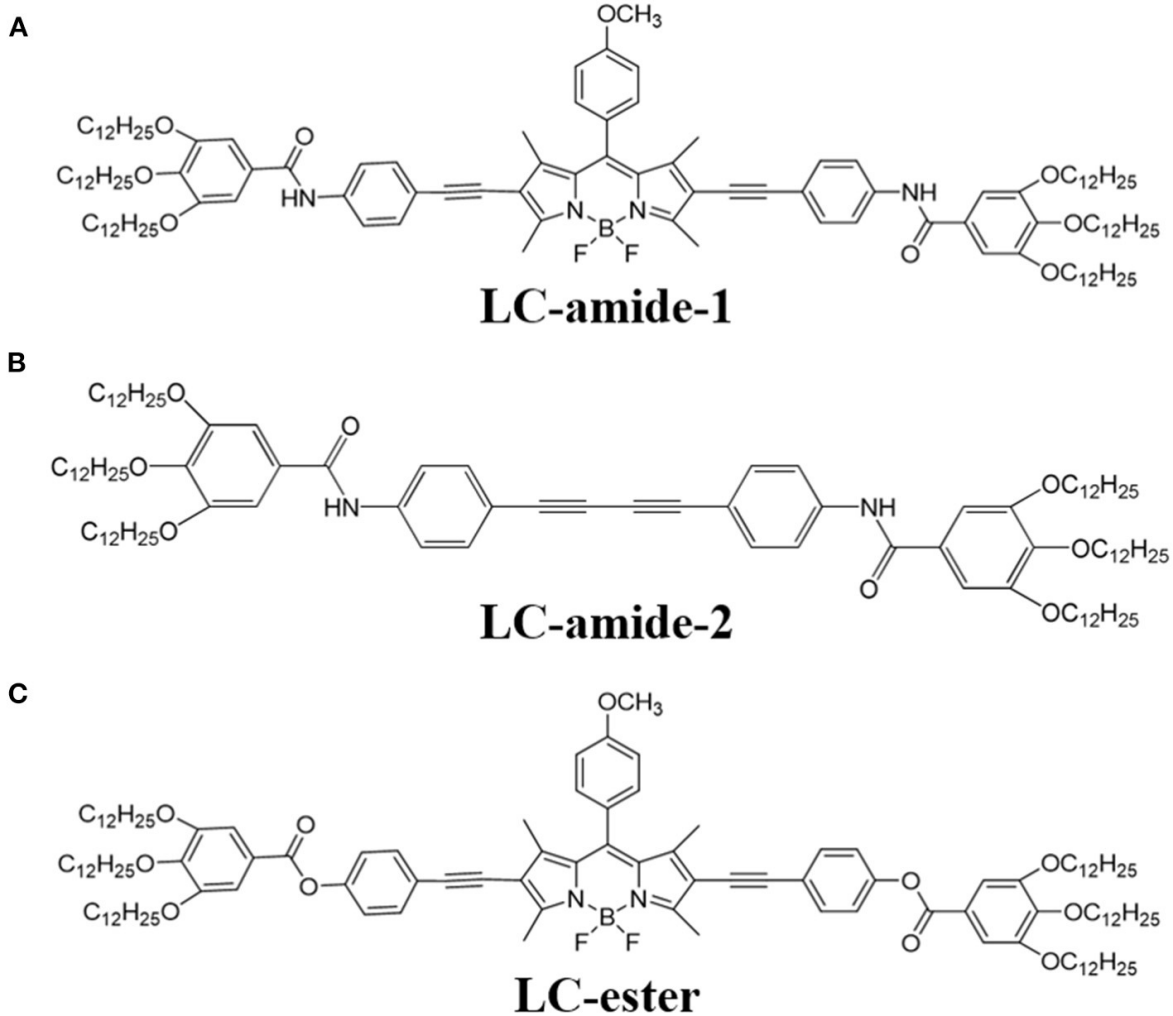
Chemical structures of **(A)** LC-amide-1, **(B)** LC-amide-2, and **(C)** LC-ester.

In our previous research (Tan S. et al., 2020), we have reported the self-assembly of three liquid crystals with similar molecular structures, namely LC-amide-1, LC-amide-2, and LC-ester. The three liquid crystal molecules all form the linear self-assembly structures, wherein the linear structures of LC-amide-1 and LC-ester are composed of rod-shaped and butterfly-shaped dimers, respectively, while LC-amide-2 molecules do not form dimers. Their uniform and regular supramolecular assembly structures provide a pathway to investigate the tribological property at nanoscale. Herein, the new self-assembly structure of LC-amide-1 at higher concentration is explicitly revealed at the 1-phenyloctane/HOPG interface by using STM under ambient conditions. Moreover, the nanotribological properties of these three liquid crystal molecules as shown in Scheme 1 are characterized by using AFM. Together with the theoretical calculation with DFT method, the nanotribological mechanism of liquid crystals is deeply explored.

## Materials and Methods

### Materials and Sample Preparation

The liquid crystal molecules were synthesized according to the reported methods (Rodle et al., 2016; Tan S. et al., 2020). 1-phenyloctane (99%) used as the solvent was purchased from J&K Chemical Ltd. (Beijing, China). All the chemicals above were used without any further purification.

The LC-amide-1, LC-amide-2, and LC-ester are dissolved in 1-phenyloctane at the corresponding concentrations. Then the self-assembly samples were prepared by depositing a droplet of the solutions (0.1 μL) onto the freshly cleaved HOPG (grade ZYA, NTMDT, Russia) surface, respectively. All the solutions and assembly samples were prepared under ambient conditions.

### Characterization and Calculation

The self-assembly structures of liquid crystals were characterized using a Nanoscope IIIA system (Bruker, Germany) under ambient conditions. And the theoretical calculations were performed using DFT-D scheme provided by DMol3 code. Further characterization and calculation details can be seen in the Supplementary Material.

### Friction Force Measurements

The microscopic friction forces of different liquid crystal systems were measured using an MFP-3D AFM (Asylum Research, America) at room temperature. To eliminate the solvent effect on the friction forces, the measurements were conducted at the gas-solid interface after the solvent was evaporated. The silicon CSG10 probe with a rectangular cantilever (nominal normal spring constant 0.3 N/m) was used. All the friction pairs are silicon probe (CSG10)—sample (assembly on HOPG). The selection of probe and loads were optimized after several attempts of friction experiments to get stable and repeatable data. It should be mentioned that the genuine normal and lateral factors need to be calibrated before each measurement. The normal photodetector sensitivity was calculated from the slope of the force curve obtained on a hard substrate. The normal spring constant was estimated from the power spectral density of thermal noise fluctuations under ambient conditions (Hutter and Bechhoefer, 1993). By scanning a commercially available TGF11 silicon grating (MikroMasch, Estonia), the lateral factor was calculated based on an improved wedge calibration method so that the voltage results can be transformed into the force values (Ogletree et al., 1996; Varenberg et al., 2003). The microscopic friction forces were measured by scanning in the perpendicular direction to the cantilever in the contact mode. The scan rate was 1 Hz, and the scan size was 160 nm × 160 nm, which is similar with the scan size of self-assembly structures in STM characterization. During the measurements, the feedback gains should be appropriate to make sure that the noise of the deflection signal was minimal while the height signal was tracked well. At least five positions were measured for each sample. For the data processing, friction values were calculated as half of the difference between the trace and retrace signals, such that the topography effect on friction can be excluded.

## Results and Discussion

### Self-Assembly of LC-Amide-1 at High Concentration

**Figure 1:**
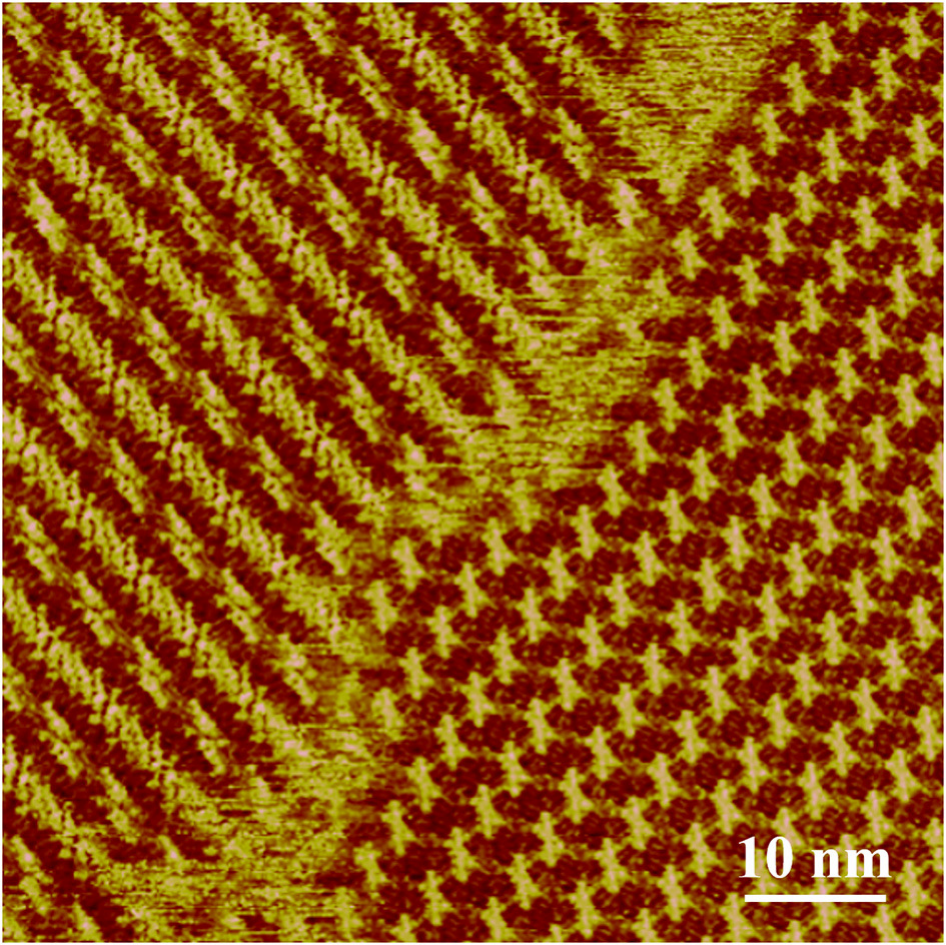
STM images of LC-amide-1 assembly structure at the HOPG/1-phenyloctane interface at higher concentration (10^−4^~10^−3^ M). Tunneling conditions: *I*_set_ = 265.5 pA, *V*_bias_ = 667.4 mV.

Our previous research has revealed the assembly structure of LC-amide-1 at 10^−4^ M, in which the dimers formed by two closed-packed molecules are aligned in parallel (Tan S. et al., 2020). However, after further investigation, we found that the assembly structure could change with the increase of concentration. As shown in Figure 1, two absolutely different assembly structures are existing simultaneously at higher concentration. Moreover, when the concentration is increased to 10^−3^ M, the uniform structure formed by new arrangement of LC-amide-1 molecules can be observed.

**Figure 2:**
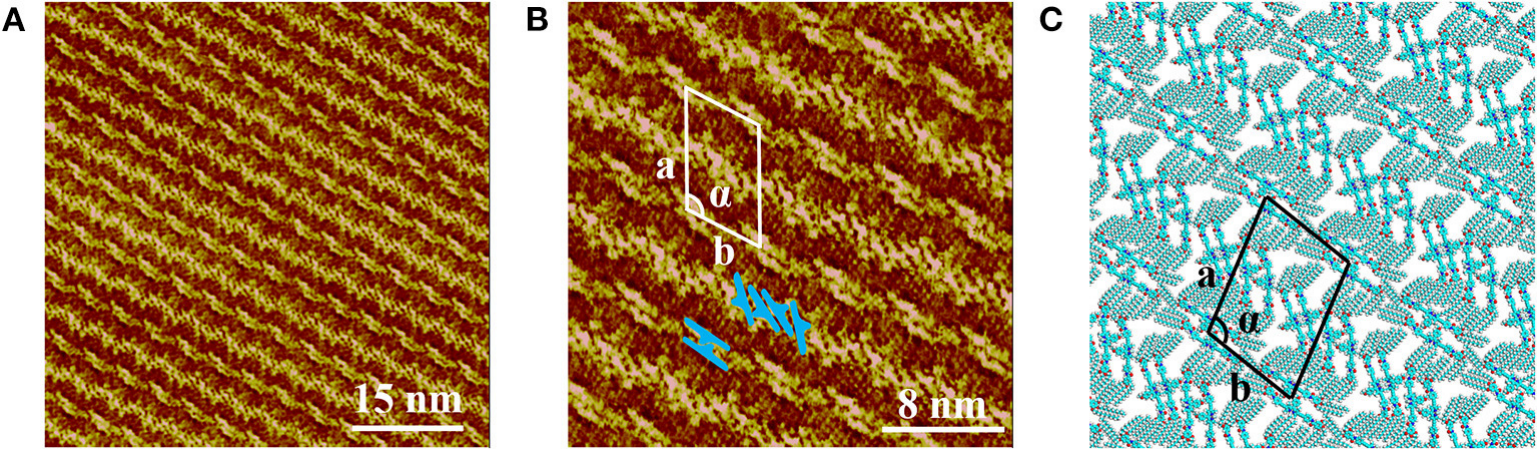
STM images of LC-amide-1 assembly structure at the HOPG/1-phenyloctane interface: **(A)** Large scale; **(B)** High resolution. Tunneling conditions: *I*_set_ = 366.2 pA, *V*_bias_ = 520.3 mV. **(C)** The simulated molecular packing structure.

Figure 2A presents the large scale STM image of LC-amide-1 assembly structure at high concentration (10^−3^ M), in which the linear arrangement of bright rods could be observed. However, the arranging patterns of two adjacent rows are quite different, in which the bright rods are loose-packed in one row and close-packed in the neighbor row. Further details of assembly structure can be observed in the high resolution STM image, as shown in Figure 2B. The loose-packed rows are lined up with dimers formed by two bright rods. The average length of bright rods is measured to be 3.7 ± 0.1 nm, which shows good agreement with the theoretical size of backbone of LC-amide-1 molecules. Therefore, it can be inferred that the bright rods correspond to the LC-amide-1 molecules. And the alkyl chains of LC-amide-1 molecules correspond to the lines distributed in the shade, which cannot be explicitly characterized due to the much lower density of electric states. Each dimer is consisted of two facing LC-amide-1 molecules, as marked with blue rods in Figure 2B. As for the close-packed rows, the LC-amide-1 molecules are closely aligned in parallel, the orientations of which are at an angle to the direction of the row, as marked with blue rods in Figure 2B likewise. The motif of uniform and ordered assembly derives from the π-π stacking interaction between corresponding benzene rings, the N–H···O hydrogen bonding between neighboring amide groups and the van der Waals interactions between the alkyl chains. The parameters of the unit cells overlaid in Figure 2B are measured as follows: *a* = 7.9 ± 0.1 nm, *b* = 5.8 ± 0.1 nm, and α = 105 ± 1°.

### Theoretical Calculation

The unit cell parameters and interaction energies of LC-amide-1 at high concentration are calculated with the DFT method based on the STM observation to further analyze the self-assembly structure. The calculated unit cell parameters (as shown in Figure 2C) are listed in Table 1, which are in accordance with the experimental results. The interaction energies between molecules are also calculated, as listed in Table 2. For comparison, the interaction energies of LC-amide-1 at low concentration, LC-amide-2, and LC-ester presented in our previous research (Tan S. et al., 2020) are also listed in Table 2. Herein, the lower interaction energy indicates the stronger interaction. Compared with three self-assembly structures in our previous research (Tan S. et al., 2020), the arrangement of LC-amide-1 molecules at high concentration is much more compact. Therefore, it can be observed that the intermolecular interaction energy of LC-amide-1 at high concentration (−535.52 kcal/mol) is the lowest. Other than the intermolecular interactions, the interactions between molecules, and the substrate also play an important role in the surface supramolecular assembly process. As shown in the second column in Table 2, the interaction energy between assembled molecules and the substrate of LC-amide-1 at high concentration (−1,704.309 kcal/mol) is much lower than all the others. It can be concluded that the π-π stacking and van der Waals interaction between LC-amide-1 molecules and HOPG at high concentration is strongest due to the much tighter alignment of molecules. Moreover, the total energy per unit area is also calculated and listed in the fourth column in Table 2, which can evaluate the interaction strength and thermodynamic stability of the assembly systems with different unit cells. It can be clearly revealed that the total energy per unit area of LC-amide-1 at high concentration (−0.479 kcal mol^−1^ Å^−2^) is lowest, which means that the interaction strength is the strongest among four assembly systems.

**Table 1:** Experimental (Expt.) and calculated (Cal.) cell parameters of LC-amide-1 at high concentration on the HOPG surface.

		**b (nm)**	**a (nm)**	**α (°)**
LC-amide-1	Expt.	5.8 ± 0.1	7.9 ± 0.1	105 ± 1°
	Cal.	5.9	8.2	105

**Table 2:** Total energies and energies per unit area of self-assemblies on the HOPG surface^a^.

	**Interactions between molecules (kcal mol^**−1**^)**	**Interactions between molecules and substrate (kcal mol^**−1**^)**	**Total energy (kcal mol^**−1**^)**	**Total energy per unit area (kcal mol^**−1**^ Å^**−2**^)**
LC-amide-1 high	−535.52	−1,704.309	−2,239.829	−0.479
LC-amide-1 low (Tan S. et al., 2020)	−149.536	−418.567	−568.103	−0.338
LC-amide-2 (Tan S. et al., 2020)	−16.254	−152.124	−168.378	−0.234
LC-ester (Tan S. et al., 2020)	−134.914	−421.721	−561.635	−0.303

a *The total energy includes the interaction energies between molecules and the interaction energies between molecules and substrate*.

### Nanotribological Properties of Liquid Crystals

**Figure 3:**
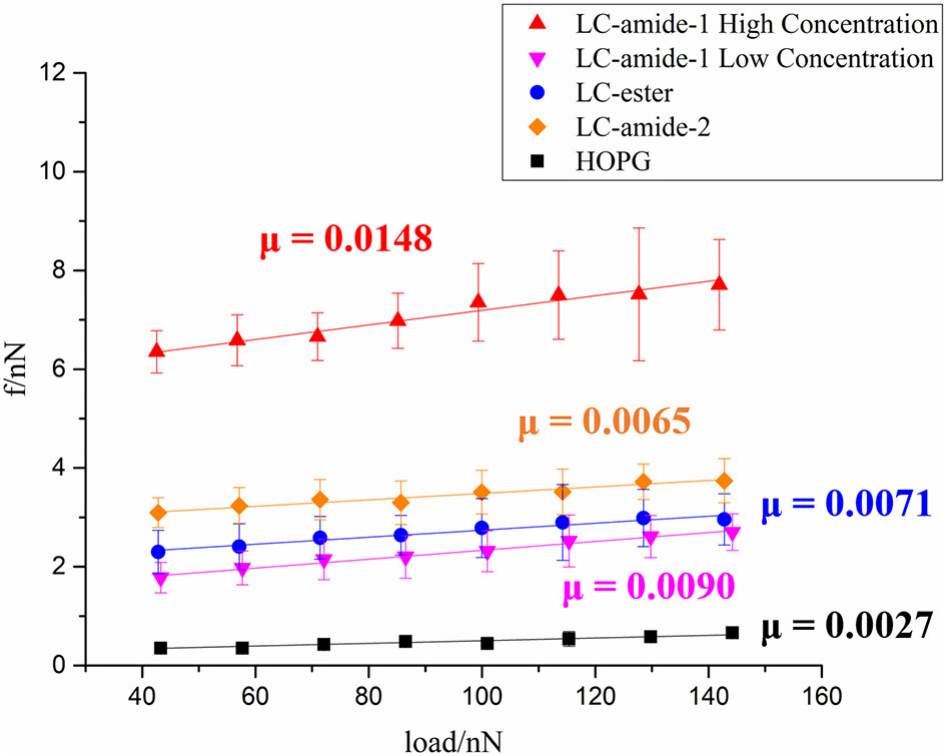
Friction force as a function of applied load of LC-amide-1 at high/low concentration, LC-amide-2, LC-ester, and HOPG.

At present, the tribological research on liquid crystals is mainly conducted at macroscale. To investigate the nanotribological performance, the friction forces of liquid crystals, including self-assembly mentioned above and three other self-assemblies in our previous research (Tan S. et al., 2020), are measured under ambient conditions by using AFM. As displayed in Figure 3, friction forces of LC-amide-1 at high concentration (10^−3^ M) and low concentration (10^−4^ M), LC-amide-2, LC-ester and HOPG are presented as a function of the applied load. For these five systems, friction forces increased linearly with the load. In comparison, the friction forces of LC-amide-1 at low concentration, LC-amide-2 and LC-ester are similar, while the friction forces of LC-amide-1 at high concentration are approximately twice larger. The friction-load data are linearly fitted to acquire friction coefficient, which can be defined as the slope of the linear fitting curve, as labeled in Figure 3. It is revealed that the friction coefficient of LC-amide-1 at high concentration is much higher than those of the other self-assembly systems, which is similar as the friction force result. As for the three other liquid crystal systems, their friction coefficients are close to each other and all below 0.01, indicating the achievement of microscale superlubricity. Specifically, the friction coefficient of LC-amide-1 at low concentration is slightly higher than that of LC-ester, while the friction coefficient of LC-amide-2 is the lowest in three systems.

**Figure 4:**
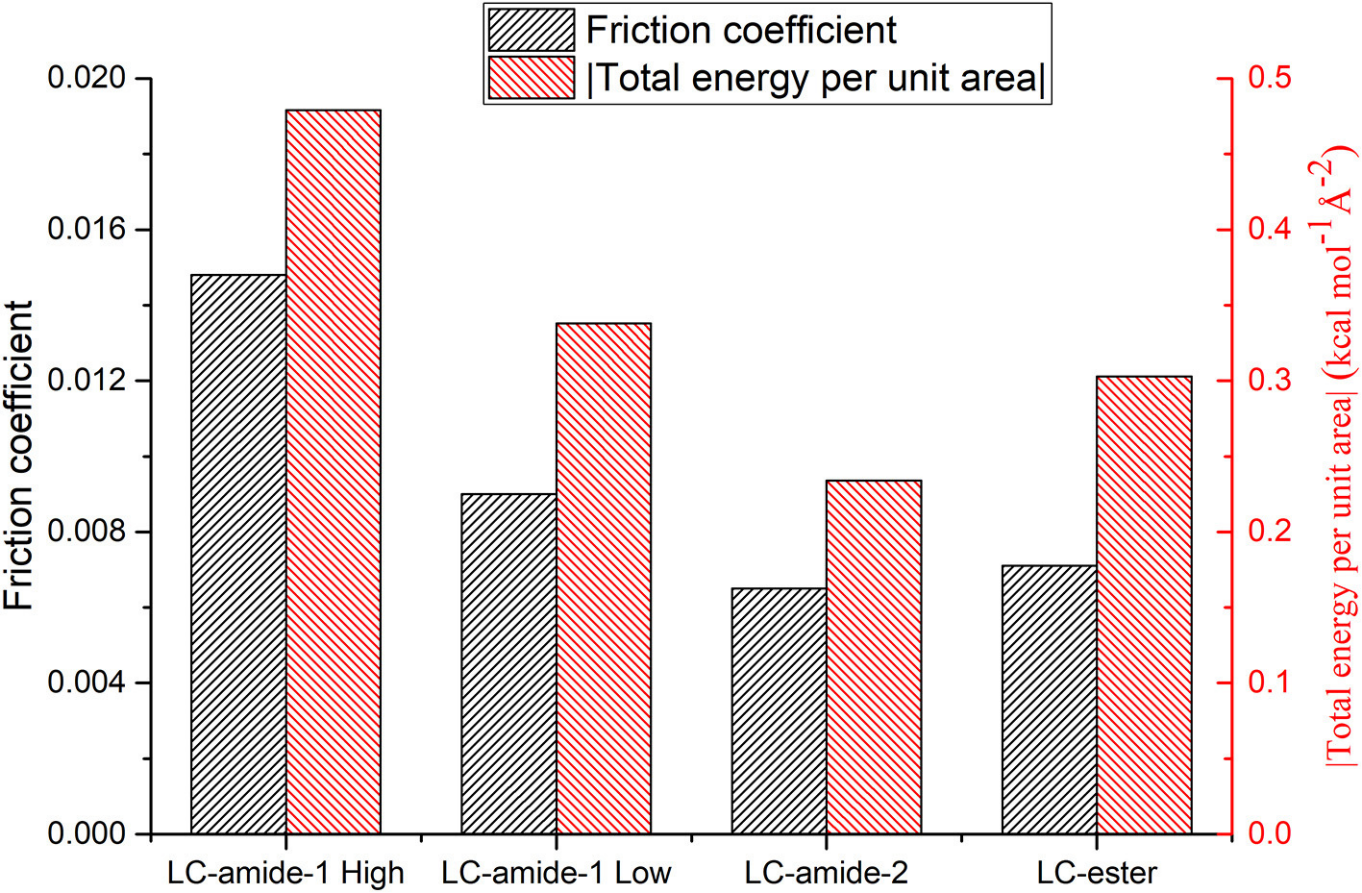
Friction coefficients and total energies per unit area (absolute value) of LC-amide-1 at high/low concentration, LC-amide-2, and LC-ester.

As mentioned above, the interaction strength of LC-amide-1 at high concentration is the strongest among all the self-assemblies. Considering that the friction coefficient of LC-amide-1 at high concentration is also the highest, it seems that the nanotribological properties are related to the interaction strength. Therefore, to better understand such correlation, the experimental friction coefficient and theoretical total energy per unit area of four liquid crystal self-assembly systems are summarized in Figure 4. It is clearly revealed that the friction coefficient is positively correlated with the absolute value of total energy per unit area. In other words, there is a positive correlation between friction coefficient and interaction strength, which agrees well with the conclusion in our previous researches (Shi et al., 2018; Tan S. C. et al., 2020). The interaction herein includes the interaction between assembled molecules and the interaction between molecules and HOPG substrate. It can be hypothesized that during the rubbing process between the AFM probe and the self-assembly, these physical interactions are ruptured by the AFM probe and regenerated rapidly, achieving a dynamic equilibrium state. The friction process can also be considered as the continuous energy barrier overcoming process of AFM probe at the interface. Therefore, the assembly with stronger interaction would display a higher friction coefficient. The microscale superlubricity of liquid crystals can only be achieved in the self-assemblies with weak interaction, while the friction coefficient of LC-amide-1 self-assembly at high concentration is higher than 0.01 because of the tighter arrangement of molecules and stronger interaction.

## Conclusion

In summary, the self-assembly structures and superlubrication properties of liquid crystals are investigated at the molecular level. The STM characterization indicates that Lc-amide-1 molecules can form a totally different assembly structures at higher concentration. More importantly, the microscale superlubricity of liquid crystals on HOPG surface is successfully achieved. The DFT calculation reveals the positive correlation between the friction coefficient and the interaction strength. From an energy point of view, the energy dissipation in frictional process derives from the continuous breaking and reforming of the physical bonds. This work of superlubricity and self-assembly of liquid crystals could enhance our understanding of the interface friction mechanisms at nanoscale and provide a promising strategy to explore the further applications of liquid crystals in lubrication.

## Data Availability Statement

The raw data supporting the conclusions of this article will be made available by the authors, without undue reservation.

## Author Contributions

ST completed the STM and AFM characterization under the guidance of YL and QZ. JT synthesized all the liquid crystals under the guidance of HX. WL completed the DFT calculation. HS provided the help of STM experiments. BT and HJ provided the help of calculation and synthesis. The manuscript was written through contributions of all authors. All authors have given approval to the final version of the manuscript.

## Conflict of Interest

The authors declare that the research was conducted in the absence of any commercial or financial relationships that could be construed as a potential conflict of interest.
