# Supplementary material for: Insight Into the Superlubricity and Self-Assembly of Liquid Crystals
Source: Front Chem. 2021 Jun 11;9:668794. doi: 10.3389/fchem.2021.668794 (PMC8226320; doi:10.3389/fchem.2021.668794)
Supplement: Supplementary file 1 [file Data_Sheet_1.docx]

Supplementary Material

# STM characterization

The samples were characterized using a Nanoscope IIIA system (Bruker, Germany) under ambient conditions. To achieve better imaging resolution of the assemblies, the measurements were carried out at the liquid-solid interface by immersing the STM tip directly into the solution. All the images were captured with the mechanically made from Pt/Ir (80:20) wires under constant-current mode. The figure captions have included the specific tunneling conditions (i.e., tunneling current and bias). The drift for all the images was calibrated using an atomic-resolution HOPG lattice as a reference. The experimental cell parameters were obtained by using the length measurement function of the STM software “*NanoScope (R) III Digital Instrument*” (Veeco Instrument Inc., America). After selecting two or three STM images, five groups of cell parameters were measured in each image and all the data were averaged to figure out the final cell parameters.

# DFT calculation

Theoretical calculations were performed using DFT-D scheme provided by DMol3 code. We used the periodic boundary conditions (PBC) to describe the 2D periodic structure on the graphite in this work. The Perdew-Burke-Ernzerhof parameterization of the local exchange correlation energy was applied in the generalized gradient approximation (GGA) to describe exchange and correlation (Perdew et al., 1996). All-electron spin-unrestricted Kohn-Sham wave functions were expanded in a local atomic orbital basis. For the large system, the numerical basis set was applied. All calculations were all-electron ones, and performed with the medium mesh. Self-consistent field procedure was done with a convergence criterion of 10^-5^ au on the energy and electron density. Combined with the experimental data, we have optimized the geometry of the adsorbates in the unit cell. When the energy and density convergence criterion were reached, we could obtain the optimized parameters and the interaction energy between adsorbates. To evaluate the interaction between the adsorbates and HOPG, we design the model system. Since adsorption of benzene on graphite and graphene should be very similar (MacLeod et al., 2015), we had performed our calculations on infinite graphene monolayers using PBC. Considering that the interaction between adsorbates and substrate was mainly van der Waals interaction, the Grimme’s dispersion corrections were adopted in our calculations. In the superlattice, graphene layers were separated by 35 Å in the normal direction. When modeling the adsorbates on graphene, we used graphene supercells and sampled the Brillouin zone by a 1x1x1 k-point mesh.

# Reference

MacLeod, J.M., Lipton-Duffin, J.A., Cui, D., De Feyter, S., and Rosei, F. (2015). Substrate Effects in the Supramolecular Assembly of 1,3,5-Benzene Tricarboxylic Acid on Graphite and Graphene. *Langmuir* 31(25)**,** 7016-7024.

Perdew, J.P., Burke, K., and Ernzerhof, M. (1996). Generalized gradient approximation made simple. *Physical Review Letters* 77(18)**,** 3865-3868.
